# Effects of crocin on T-bet/GATA-3 ratio, and miR-146a and miR-106a expression levels in lung tissue of ovalbumin-sensitized mice 

**DOI:** 10.22038/IJBMS.2022.65622.14433

**Published:** 2022-10

**Authors:** Mohammad Reza Aslani, Zahra Jafari, Reza Rahbarghazi, Jafar Rezaie, Aref Delkhosh, Mahdi Ahmadi

**Affiliations:** 1 Lung Diseases Research Center, Ardabil University of Medical Sciences, Ardabil, Iran; 2 Applied Biomedical Research Center, Mashhad University of Medical Sciences, Mashhad, Iran; 3 Student Research Committee, Tabriz University of Medical Sciences, Tabriz, Iran; 4 Department of Physiology, Faculty of Medicine, Tabriz University of Medical Sciences, Tabriz, Iran; 5 Drug Applied Research Center, Tabriz University of Medical Sciences, Tabriz, Iran; 6 Stem Cell Research Center, Tabriz University of Medical Sciences, Tabriz, Iran; 7 Solid Tumor Research Center, Research Institute for Cellular and Molecular Medicine, Urmia University of Medical Sciences, Urmia, Iran

**Keywords:** Asthma, GATA-3, microRNA-106a, microRNA-146a, T-box transcription factor

## Abstract

**Objective(s):**

Although various studies have revealed the beneficial effects of crocin (derived from saffron), such as anti-inflammatory, anti-cancer, antioxidant, and immune modulator, however, its exact mechanism is unknown. The present study aimed to investigate the effect of crocin on the expression ratio of T-bet/GATA-3 as an indicator of altered immune responses in the lung tissue of ovalbumin (OVA)-sensitized mice. In addition, the effect of crocin on the expression level of miR-146a and miR-106a in the lung tissue OVA-sensitized mice was investigated.

**Materials and Methods:**

Mice were randomly divided into five groups (n=6): Control; OVA, OVA + Crocin 25, OVA + Cro 50, and OVA + Cro100 groups. Crocin was administrated intraperitoneally at doses of 25, 50, and 100 mg/kg for five consecutive days. One day after asthma induction, animals were euthanized, and lungs were sampled for pathological and gene expression analysis.

**Results:**

OVA-sensitization led to increased inflammation and histopathological changes in the lung tissue of mice. In addition, GATA-3 expression increased (*P*<0.001) and T-bet expression decreased (*P*<0.001) in OVA-sensitized groups. The T-bet/GATA3 ratio was also reduced markedly in asthma groups (*P*<0.001). Furthermore, increased expression of miR-146a and miR-106a levels was evident in the lung tissue of OVA-sensitized mice (*P*<0.001 for both). Intervention with high concentrations of crocin (50 and 100 mg/kg) significantly reduced airway inflammation, GATA-3 expression, miR-146a expression, and miR-106a expression and corrected the T-bet/GATA-3 ratio (*P*<0.05 to *P*<0.001).

**Conclusion:**

Treatment with crocin led to a decrease in the severity of lung inflammation in OVA-sensitized mice, which is probably through the reduction of the T-bet/GATA-3 ratio, and mir-146a and mir-106a expression level.

## Introduction

*Asthma* is a chronic inflammatory disease associated with various symptoms, including airway obstruction and hyper-responsiveness (1). Inflammation in asthma is caused by increased and decreased immune responses of Th_2_ and Th_1_ cells, respectively (2). Increased Th_2_ cell activity led to elevated IL-4 levels, and decreased Th_1_ cell activity led to decreased INF-γ levels, indicating altered immune responses in patients with asthma (2). GATA-3 transcription factor acts as the primary regulator of Th_2_ differentiation and increases its production of inflammatory cytokines such as IL-4, IL-5, and IL-13 (3, 4). On the other hand, transcription factor T-bet, a member of the T-box family, plays a vital role in the differentiation and effector function of Th_1_ cells (5, 6). An imbalance between T-bet and GATA-3 transcription factors has recently been reported in bronchial asthma (7). Therefore, one of the treatment goals in patients with asthma is to correct the Th_1_/Th_2_ imbalance, so increasing the Th_1_/Th_2_ ratio is an essential indicator of improving immune responses in asthmatic patients (8, 9).

Micro-RNAs (miRs) are small non-coding RNAs that regulate gene expression after transcription by inhibiting mRNA or inducing its degradation (10, 11). Recently, miRs have been shown to play a key role in regulating immune and inflammatory responses, lymphocyte activation, and eosinophil evolution (12). However, the role of many of them in patients with asthma is not well understood. Elevated miR-146a levels have been observed in patients with asthma (13). Interestingly, in obese ovalbumin-sensitized rats, increased expression level of miR-146a was more evident in lung tissue (13). Increased expression of miR-106a has also been reported in experimental asthma. By inhibiting miR-106a activity, a reduction in airway inflammation and mucous secretions occurred in the lung tissue of OVA-sensitized mice by increasing IL-10 production and decreasing the infiltration of inflammatory cells into the airways (14, 15). Therefore, another therapeutic goal in patients with asthma can be to focus on the activity of miRs. 

The effectiveness of medicinal plants in chronic inflammatory diseases has been demonstrated in various human and animal studies (16-19). One recommended herbal medicine for asthma patients is saffron and its active ingredient crocin (20, 21). Experimental and clinical studies have revealed the effectiveness of crocin in chronic inflammatory diseases such as rheumatoid arthritis, heart disease, central nervous system, kidney disease, and lung disease (21-25). Although various mechanisms have been reported for the effectiveness of crocin in asthma conditions, such as decreased Th_2_ lymphocyte activity, modulated expression of endoplasmic reticulum stress genes, and improved oxidant/antioxidant imbalance (21, 22), the exact mechanism is not well understood. Therefore, the present study aimed to investigate the effect of crocin on the expression ratio of T-bet/GATA-3 as an indicator of altered immune responses in the lung tissue of ovalbumin-sensitive mice. The current study also evaluated the effects of crocin on miR-146a and miR-106a expression.

## Materials and Methods

***Chemicals***

Quantitative enzyme-linked immunosorbent assay (ELISA) kits for OVA-sensitive IgE were obtained from Crystal Day (Shanghai, China). The crocin standard (>98%) was obtained from Sigma-Aldrich (St. Louis, MO, USA). OVA was obtained from Sigma-Aldrich (St. Louis, MO, USA). Aluminum hydroxide gel was obtained from Thermo Fisher (Waltham, MA, USA). All RT-PCR chemicals were obtained from Yekta Tajhiz Co. (Tehran, Iran). All other chemicals used in the study were of analytical grade.

***Experimental design***

This study used 30 adult male mice weighing 25 to 30 g. The animals were obtained from the Animal House of Tabriz University of Medical Sciences and adapted to the environment for one week. A temperature of 22±2 °C, a light-dark cycle of 12 hr: 12 hr, and free access to water and food were provided for all animals during the study. All animal-related interventions were performed after approval by the TBZMED ethics committee (IR.TBZMED.VCR.REC.1399.059). 

**Figure 1:**
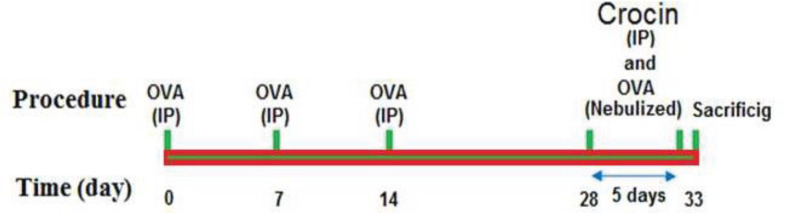
Experimental design flow chart and treatment with saline and crocin (last 5 days of the model). OVA; Ovalbumin, IP; Intraperitoneally

The animals were divided into five groups (n = 6 in each group) according to Figure 1. For sensitization with ovalbumin (OVA), the model of previous studies was used (21). In summary, 10 μg of OVA and 2 mg of aluminum hydroxide (Al (OH)_3_) were injected intraperitoneally on days 0, 7, and 14. From the 28th to the 32nd day, the animals were then exposed to 1% OVA aerosol through the nose for 30 min. In the control group, the animals received normal saline instead of OVA. In the intervention groups, one hour before the challenge with OVA, the animals were treated with crocin (IP).

***Bronchoalveolar lavage fluid (BALF) collection***

In order to collect BALF of animals after anesthesia with ketamine and xylazine (100 mg/kg and 10 mg/kg, IP, respectively), tracheal cannulation was performed. Sample collection was performed by injecting and aspirating 0.5 ml of phosphate buffer saline (PBS) (three times). The supernatant prepared from the BALF sample was used for total white blood cell (WBC) and differential cell count (26).

***Total and differential white blood cell (WBC) count***

Total WBC count was performed using a hemocytometer and Wright-Giemsa staining. Differential cell count was performed by a light microscope with ×400 magnification and following the standard protocol (26).

***Tissue sampling and protein measurement***

Right lungs were frozen in liquid nitrogen and stored at -70 °C until OVA-sensitive IgE was measured. To prepare a supernatant, tissue samples were weighed and homogenized in PBS (pH 7.2–7.4) and centrifuged for 20 min at 4 °C at 3000 rpm (21). According to the manufacturer’s instructions, OVA-specific IgE (µg/gram total protein) was measured using mouse ELISA commercial kits (Crystal Day, Shanghai, China). 

***Real-time polymerase chain reaction***

We performed real-time PCR analysis to evaluate GATA-3 and T-bet mRNA expression levels and miR-146a and miR-106a (10, 27). Table 1 shows the locked nucleic acid (LNA) forward and reverses mRNA’s primer sets (Exiqon). The PCR products were normalized with β-actin genes for mRNA samples. Results were expressed as fold change versus controls.

***Pathological assessment***

Isolated left lung tissue was fixed in 10% neutral buffered formalin (37%, Merck, Germany) and embedded with paraffin blocks. The paraffin blocks were then cut to 4 μm, stained with hematoxylin-eosin, and evaluated under a light microscope. Pathological changes included a detachment of epithelium, bronchioles infiltration of lymphocytes, and interstitial tissue pneumonia. Scoring for each pathological lung change was identified from 0 to 3 as follows: 0= normal; 1= patchy injury, 2= local injuries, and 3= scattered injuries (27, 28). 

***Statistical analysis***

In the current study, results are reported as mean ±SEM. Comparisons among different groups were performed using variance (ANOVA) with Tukey-Kramer *post hoc test*. In addition, Kruskal-Wallis statistical test was used to analyze the pathological results. *P*<0.05 was considered the significance level.

## Results

***Effects of crocin on the BALF cell infiltration***

**Figure 2:**
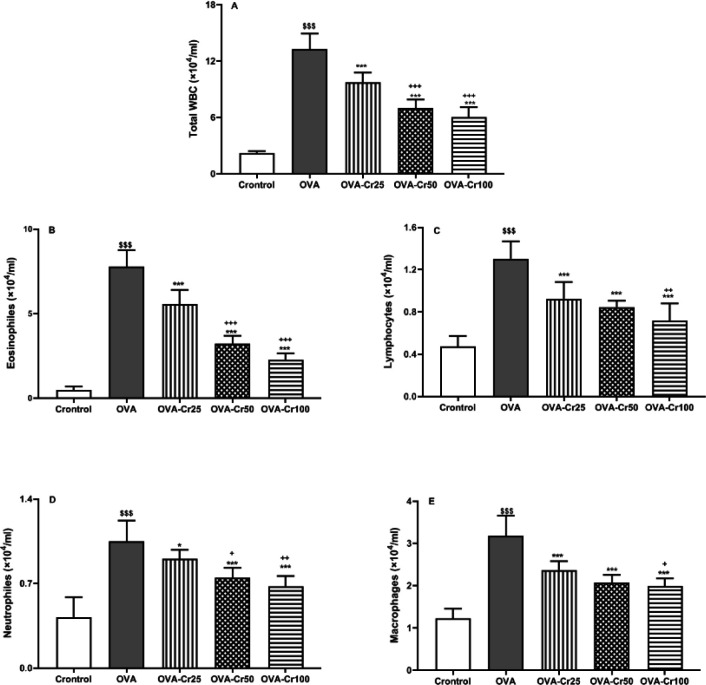
Total WBC (A), eosinophil (B), lymphocyte (C), neutrophil (D), and macrophage (E) counts in the BALF of the control, asthmatic (OVA), OVA- crocin 25 mg/kg (OVA-Cr25), OVA- crocin 50 mg/kg (OVA-Cr50), and OVA-crocin 100 mg/kg (OVA-Cr100) groups. Data are shown as mean±SEM. $$$: *P*<0.001 control vs OVA group. *: *P*<0.05 and ***: *P*<0.001 OVA group vs crocin treated groups. +: *P*<0.05, ++: *P*<0.01, and +++: *P*<0.001 OVA-Cr25 group vs OVA-Cr50 and OVA-Cr100 groups. &&: *P*<0.01 OVA-Cr50 group vs OVA-Cr100 group. For each group, n = 6. Comparisons between groups were made using the ANOVA test

The total number of WBCs in the sensitized group was significantly higher in comparison with the control group (*P*<0.001). Instead, the number of leukocytes in the crocin-treated groups was significantly lower than in the OVA group (*P*<0.001 for all) (Figure 2A).

Here we reported that in the OVA group, levels of all inflammatory cells, including eosinophils, lymphocytes, neutrophils, and macrophages were significantly higher than in control animals (*P*<0.001 for all cases, Figure 2B-E). The significant improvement in the levels of all inflammatory cells in the treated groups (OVA-Cr25, OVA-Cr50, and OVA-cr100) was seen in comparison with the OVA group (*P*<0.001 for all cases, Figures 2B-E). However, total and differential leucocyte count indices in the BAL samples of all treated groups were still higher than in the healthy animals. 

***Effect of crocin on GATA-3 and T-bet expression levels in the lung tissue***

**Figure 3:**
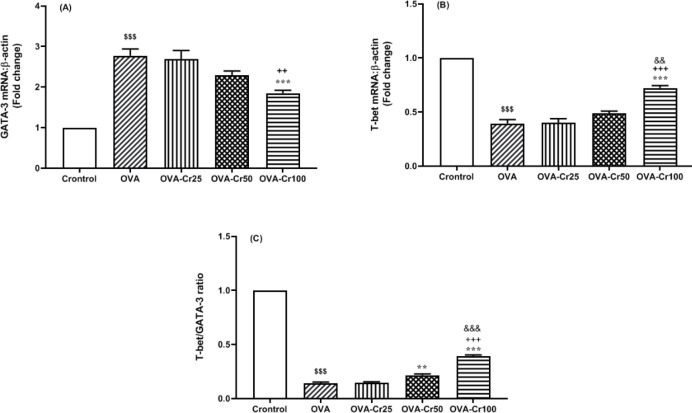
Lug tissue GATA-3 and T-bet gene expression levels. Mean+SEM of A) GATA-3, B) T-bet, and C) T-bet/GATA-3 ration. Abbreviations are the same as Figure 2. $$$: *P*<0.001 control vs OVA group. **: *P*<0.01 and ***: *P*<0.001 OVA group vs crocin treated groups. ++: *P*<0.01 and +++: *P*<0.001 OVA-Cr25 group vs OVA-Cr50 and OVA-Cr100 groups. &&: *P*<0.01 and &&&: *P*<0.001 OVA-Cr50 group vs OVA-Cr100 group. For each group, n = 6. Comparisons between groups were made using the ANOVA test

OVA-sensitization increased the expression level of GATA-3 in the lung tissue of mice, which crocin at a concentration of 100 mg/kg significantly prevented (*P*<0.01, Figure 3A). The inhibitory effect of crocin at 100 mg/kg was significantly higher than that of 50 mg/kg (*P*<0.05). On the other hand, the expression of T-bet mRNA level in lung tissue of OVA-sensitized mice was significantly reduced compared with the control group (*P*<0.001 for all) (Figure 3B). Crocin at a 100 mg/kg concentration resulted in a significant increase in T-bet expression levels (*P*<0.001, Figure 3B). Furthermore, the results showed that the T-bet/GATA-3 ratio was significantly reduced in the OVA-sensitized groups compared with the control group (Figure 3C). Intervention with 50 and 100 mg/kg crocin concentrations prevented T-bet/GATA-3 reduction. 

***Effect of crocin on miR-146a and miR-106a expression levels in the lung tissue***

**Figure 4:**
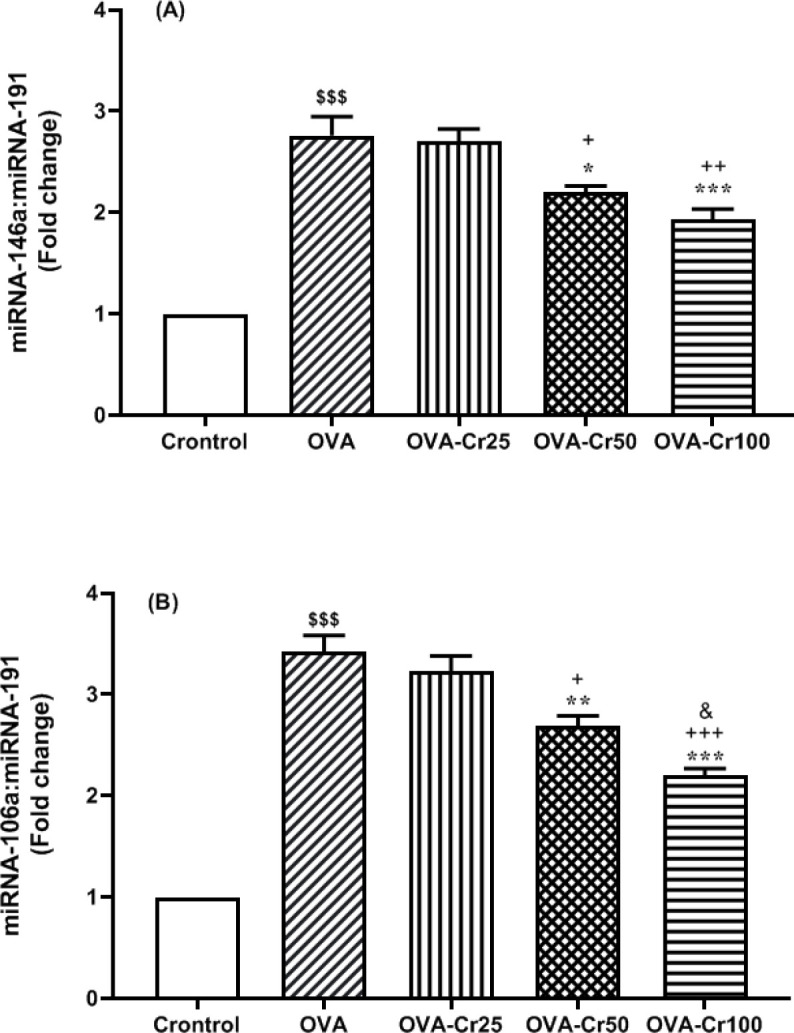
Lug tissue miR-146a and miR-106a gene expression levels. Mean+SEM of A) miR-146a and B) miR-106a expression levels. Abbreviations are the same as Figure 2. $$$: *P*<0.001 control vs OVA group. *: *P*<0.05, **: *P*<0.01, and ***: *P*<0.001 OVA group vs crocin-treated groups. +: *P*<0.05, ++: *P*<0.01, and +++: *P*<0.001 OVA-Cr25 group vs OVA-Cr50 and OVA-Cr100 groups. &: *P*<0.05 OVA-Cr50 group vs OVA-Cr100 group. For each group, n=6. Comparisons between groups were made using the ANOVA test

The elevated expression level of miR-146a was significantly observed in the OVA-sensitized groups compared with the control group (*P*<0.001 for all). Concentration-dependent crocin-treatment (50 and 100 mg/kg) inhibited the increased expression of miR-146a (*P*<0.05 and *P*<0.01, respectively), which had a higher effect on crocin 100 than 50 mg/kg (*P*<0.05) (Figure 4A). The OVA-sensitization effects were also significantly associated with enhanced miR-106a expression levels in mice lung tissue compared with the control group (*P*<0.001 for all). Crocin 50 and 100 mg/kg significantly inhibited the increase in miR-106a expression compared with the OVA group (*P*<0.01 and *P*<0.001, respectively) (Figure 4B). 

***Effects of crocin on the OVA-specific IgE protein levels in the lung tissue***

**Figure 5:**
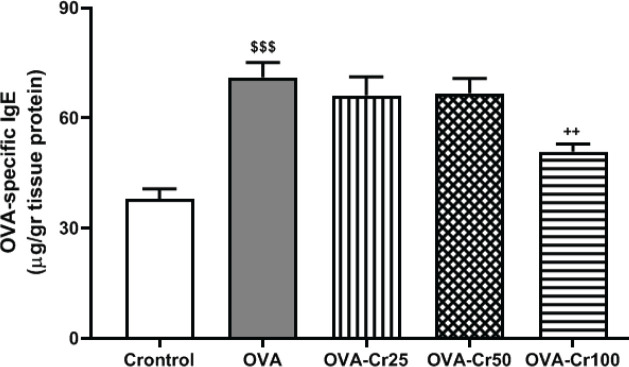
Lug tissue protein levels of OVA-specific IgE. Values are expressed as mean+SEM. Abbreviations are the same as Figure 2. $$$: *P*<0.001 control vs OVA group. ++: *P*<0.01 OVA group vs crocin-treated groups. For each group, n=6. Comparisons between groups were made using the ANOVA test

OVA-sensitization resulted in increased OVA-specific IgE protein levels (69.17 ± 9.92 µg) compared with the control group (36.50 ± 6.17 µg) (*P*<0.001, Figure 5). Crocin at a concentration of 100 mg/kg significantly reduced OVA-specific IgE protein levels (49.83 ± 5.18 µg) compared with the OVA group (*P*<0.01), while concentrations of 25 and 50 mg/kg had no effect (Figure 5).

***Effects of crocin on lung tissue pathological changes***

**Figure 6:**
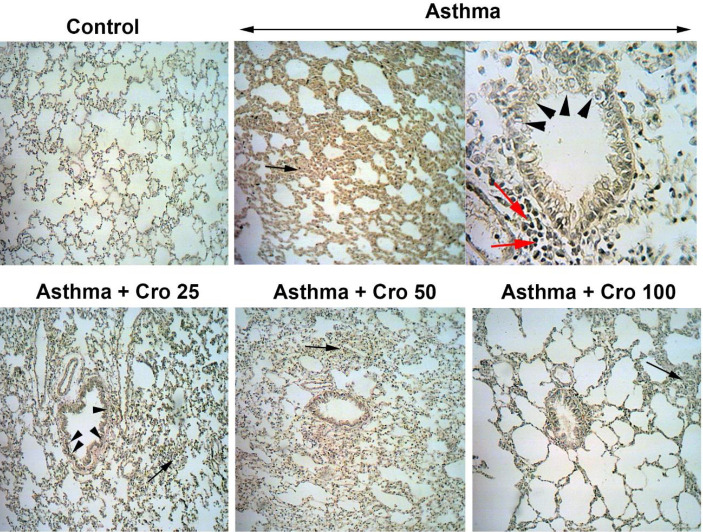
Pathological changes of pulmonary tissue (magnification=X40) in the control group, OVA-sensitized group (OVA), and crocin-treated groups (OVA-Cr25, OVA-Cr50, and OVA-Cr100). The asterisk indicates interstitial pneumonia, the headache sign indicates epithelial detachment, and the red arrow sign indicates lymphocyte infiltration

The pathological findings revealed that the severity of changes such as lymphocyte infiltration, epithelial layer detachment, and interstitial tissue pneumonia (interalveolar septal thickening) was significantly higher in the OVA-sensitized mice than in the control group (*P*<0.001 for all) (Table 2 and Figure 6). Intervention with 50 and 100 mg/kg of crocin concentrations significantly reduced histopathological changes (Table 2). 

**Table 1:** Primer sequence

Gene	Forward	Reverse
β-actin	GGCACCACACCTTCTACAATG	GGGGTGTTGAAGGTCTCAAAC
T-bet	GGGTGGACATATAAGCGGTTC	AGCAGCCGCTCACGGAG
GATA-3	GCGGGCTCTATCACAAAATGA	GCCTTCGCTTGGGCTTAAT

WBC: white blood cell; BALF: Bronchoalveolar lavage fluid

OVA: Ovalbumin

OVA: Ovalbumin

OVA: Ovalbumin

**Table 2.** Lung pathological scales in different groups

Pathological results	Scores in groups (for each group, n=6) (Minimum-maximum)				
	Control	OVA	OVA-Cr25	OVA-Cr50	OVA-Cr100
Detachment of epithelium	(0-0)	(2-3)$$$	(1-3)$$$	(1-3)$$$	(0-2)++
Lymphocytes bronchioles infiltration	(0-1)	(2-3)$$$	(2-3)$$$	(1-3)$$+	(1-3)$$+
Interstitial pneumonia	(0-1)	(2-3)$$$	(2-3)$$$	(1-3)$$$+	(0-2)$+

## Discussion

This study showed that OVA-sensitization led to an increase in inflammatory cells in the airways of mice and histopathological changes in the lung tissues that were prevented by treatment with crocin, especially at high concentrations. In addition, in OVA-sensitized mice, increased expression of GATA-3 and decreased T-bet occurred in lung tissue, and crocin with high concentrations (50 and 100) significantly prevented their changes. Finally, increased expression of mir-146a and mir-106a in lung tissue of OVA-sensitized mice was suppressed by crocin intervention.

*Asthma* is an inflammatory disease characterized by AHR, the production of Th2 inflammatory cytokines (IL-4, IL-5, and IL-13), and the increased infiltration of inflammatory cells into the airways that results from the activation of Th2 cells (29, 30). In addition, histopathological changes in asthma patients are evident, including mucus secretion, airway smooth muscle hypertrophy, lymphocyte infiltration, epithelial detachment, interstitial tissue pneumonia, subepithelial fibrosis, and goblet cell hyperplasia (31, 32). 

One of the causes of AHR in asthmatic patients is the infiltration of eosinophils in the respiratory airways, which affects the severity of the disease by producing Th2 inflammatory cytokines (5). The present study results identified that increased levels of eosinophils, macrophages, and neutrophils were evident in the BALF samples of OVA-sensitized mice. In addition, OVA-induced histopathological changes such as lymphocyte infiltration, epithelial detachment, and interstitial tissue pneumonia also occurred, indicating induction of the asthma model in this study. Accordingly, one of the therapeutic goals in patients with asthma may be to prevent the eosinophil requirement (5). 

The anti-inflammatory, antioxidant, and anti-allergic effects of saffron and its active ingredients (crocin, crocetin, and safranal) have been reported in various animal and human studies (33, 34). The anti-inflammatory and immunomodulatory activities of saffron and crocin on leukocytes and lymphocyte cells have been demonstrated under OVA-sensitized animals (34). This study revealed that crocin prevented airway eosinophilia and lung inflammation. In addition, in previous studies, the reducing effects of saffron and crocin on Th_2_ cytokine levels (IL-4, IL-5, and IL-13) also have been reported (34). The results suggest that crocin had induced anti-inflammatory effects in OVA-sensitization status. 

In asthma, Th_1_/Th_2_ imbalance has been shown to play a critical role in the pathogenesis of the disease (35). CD4 + T cells are divided into Th_1_ and Th_2_ based on functional differences and the type of cytokines produced (35). Two transcription factors, GATA-3 and T-bet, determine the differentiation of T cells to Th_2_ and Th_1_, respectively (5). IL-4 and INF-γ levels in asthma patients have been associated with the T-bet/GATA-3 ratio, indicating an immune imbalance in asthma conditions (35). In addition, a decrease in the T-bet /GATA3 ratio has been shown in most animal and human studies (36). Another therapeutic goal in asthma patients could be to modify the T-bet/GATA-3 ratio. The results showed that GATA-3 and T-bet expression levels were significantly increased and decreased in OVA-sensitized mice. Interestingly, there was a significant decrease in the T-bet/GATA-3 ratio in OVA-sensitized mice, indicating a significant increase in GATA-3 expression level. The current study results were consistent with the previous study results, which showed a more significant increase in GATA-3 compared with a decrease in T-bet in asthmatic mice (5). In fact, the role of GATA-3 in controlling Th_1_/Th_2_ differentiation appears to be greater than that of T-bet, as the direct effect of GATA-3 on IL-5 expression has been reported (5). Crocin treatment prevented T-bet/GATA-3 ratio imbalance, especially at high concentrations. Although there are not many findings regarding the role of crocin on the T-bet/GATA-3 ratio, a recent study by Hosseinzadeh *et al.* reported the modifying effects of crocin on ConA-treated human lymphocyte proliferation (37). Crocin-treated cells showed slightly lower T-bet/GATA-3 and INF-γ/IL-4 ratios than untreated cells (37). In another study, the effects of crocin on GATA-3 and T-bet expression in mononuclear cells of patients with osteoarthritis (OA) were investigated (38). The results revealed that crocin treatment significantly increased GATA-3 expression in mononuclear cells (38). Contradictory results of crocin effects in inflammatory diseases need further study. 

Various studies have shown that mirRs are involved in immune regulation and play a vital role in the therapeutic aspects of immune-related diseases (39). Elevated miR-146a and miR-106a have been reported in OVA-sensitized mice (13, 14). The present study results revealed that OVA-sensitization increased the expression of miR-146a and miR-106a in the lung tissue of mice, which was consistent with previous findings (13, 14). Crocin treatment reduced the expression of miR-146a and miR-106a levels markedly at high concentrations. Inhibition of miR-146a and miR-106 expression in an asthmatic animal model has improved disease severity (40). MiR-146a may exacerbate the disease in asthma patients by increasing IL-1β production and miR-106a by decreasing IL-10 production (13, 14). Interestingly, miR-146a has been reported to play a dual role in asthma. Some studies have shown miR-146a as an anti-inflammatory factor, the reduction of which leads to an increase in neutrophil migration (41). On the other hand, miR-146a expression increases in response to IL-17A, TNF-α, and IL-4, which indicates its negative feedback effects on inflammatory cells such as neutrophils (41, 42). It seems that the results of the current study, on the other hand, confirmed the anti-inflammatory effects of miR-146a in conditions of ovalbumin sensitization. Intervention with crocin may have prevented the increase in miR-146a expression by reducing the number of leukocytes and inflammatory cytokines. In relation to the role of mir-106, it has also been shown that its increased expression was caused by IL-4, which led to inhibitory effects on the expression of Th_2_ cells (43). The results of the current study also showed that sensitization with ovalbumin led to an increase in the expression of miR-106a, the levels of which treatment with crocin reduced . In fact, reducing the severity of the disease affected the expression of miR-106a

Little is known about the association between mirRs and transcription factors, especially in patients with asthma. In a study, Saki *et al*. showed that ectopic expression of miR-146a increased the expression of various transcription factors such as PU.1, c-Fos, CCAAT/enhancer-binding protein alpha (C/EBPα), GATA3, Foxp3, and Runx1 in lymphoblastic cells (44). The current study results were in line with the findings of the Saki study. Although the present study did not directly evaluate the effects of miR-146a on GATA-3 expression level, a significant positive correlation between GATA-3 and miR-146a reflects the role of miR-146a in GATA-3 expression, which requires further studies. On the other hand, the role of miR-146a in T-bet expression was observed as an increased expression in peripheral blood mononuclear cells in patients with acute coronary syndrome (45), which was different from the findings of the current study. The results revealed a significant negative association between miR-146a and T-bet expression in lung tissue of OVA-sensitized mice. The differences between the current study’s findings and the previous study may be due to differences like the diseases that have affected immune responses. 

GATA-3 and IL-4, which are two specific markers of Th2 cells, as well as miR-106a were revealed to be significantly increased in Th2 cells and unchanged or less expressed in Th17 cells, which actually confirms the specific differentiation (46). The relationship between miR-106a and the transcription factors T-bet and GATA-3 is not clear. Based on the findings of the current study, at least in part, it can be inferred that miR-106 may have indirect effects on the expression and function of the above transcription factors, which requires further studies.

## Conclusion

The study had some limitations. First, the study results evaluated the expression of genes and did not specify their post-translation changes. It is better to study the change levels of target genes in future studies. Second, cell line studies should be designed in terms of mechanism evaluation to evaluate the association between miR-146a and miR-106a with transcription factors GATA-3 and T-bet.

In summary, the present study results revealed changes in the expression of transcription factors associated with Th_1_ and Th_2_ cells (T-bet and GATA-3, respectively) occurring in OVA-sensitized mice*, *which treatment with crocin significantly prevented. In addition, increased expression of miR-146a and miR-106a was observed in the lung tissue of OVA-sensitized mice, which was prevented with crocin intervention. Interestingly, there was a significant positive correlation between miR-146a and miR-106a with GATA-3 and a significant negative correlation with T-bet, at least in part, reflecting the role of miRs in the expression of transcription factors.

## Authors’ Contributions

MRA and MA Helped with proposal writing, literature search, data collection, interpretation of data, analysis of data, review of manuscript, and manuscript preparation. ZJ, RR, JR, and AD^:^ Provided proposal writing, draft preparation, review of manuscript, and analysis of data.

## Funding

This study was supported by a grant (IR.TBZMED.VCR.REC.1399.059) from the Stem Cell Research Center of Tabriz University of Medical Sciences.

## Conflicts of Interest

The authors have declared that there are no conflicts of interest.
